# Innovative trial approaches in immune-mediated inflammatory diseases: current use and future potential

**DOI:** 10.1186/s41927-021-00192-5

**Published:** 2021-07-02

**Authors:** Michael J. Grayling, Theophile Bigirumurame, Svetlana Cherlin, Luke Ouma, Haiyan Zheng, James M. S. Wason

**Affiliations:** 1 Population Health Sciences Institute, Newcastle University, Baddiley-Clark Building, Richardson Road, Newcastle upon Tyne, NE2 4AX UK; 2 MRC Biostatistics Unit, University of Cambridge, Cambridge, UK

**Keywords:** Adaptive design, Basket design, Bayesian design, Composite endpoint, High-dimensional data, Routinely collected data, SMART trial, Umbrella design

## Abstract

**Background:**

Despite progress that has been made in the treatment of many immune-mediated inflammatory diseases (IMIDs), there remains a need for improved treatments. Randomised controlled trials (RCTs) provide the highest form of evidence on the effectiveness of a potential new treatment regimen, but they are extremely expensive and time consuming to conduct. Consequently, much focus has been given in recent years to innovative design and analysis methods that could improve the efficiency of RCTs. In this article, we review the current use and future potential of these methods within the context of IMID trials.

**Methods:**

We provide a review of several innovative methods that would provide utility in IMID research. These include novel study designs (adaptive trials, Sequential Multi-Assignment Randomised Trials, basket, and umbrella trials) and data analysis methodologies (augmented analyses of composite responder endpoints, using high-dimensional biomarker information to stratify patients, and emulation of RCTs from routinely collected data). IMID trials are now well-placed to embrace innovative methods. For example, well-developed statistical frameworks for adaptive trial design are ready for implementation, whilst the growing availability of historical datasets makes the use of Bayesian methods particularly applicable.

To assess whether and how these innovative methods have been used in practice, we conducted a review via PubMed of clinical trials pertaining to any of 51 IMIDs that were published between 2018 and 20 in five high impact factor clinical journals.

**Results:**

Amongst 97 articles included in the review, 19 (19.6%) used an innovative design method, but most of these were relatively straightforward examples of innovative approaches. Only two (2.1%) reported the use of evidence from routinely collected data, cohorts, or biobanks. Eight (9.2%) collected high-dimensional data.

**Conclusions:**

Application of innovative statistical methodology to IMID trials has the potential to greatly improve efficiency, to generalise and extrapolate trial results, and to further personalise treatment strategies. Currently, such methods are infrequently utilised in practice. New research is required to ensure that IMID trials can benefit from the most suitable methods.

**Supplementary Information:**

The online version contains supplementary material available at 10.1186/s41927-021-00192-5.

## Background

Immune-mediated inflammatory diseases (IMIDs) consist of many distinct conditions that share common inflammatory pathways. They range in prevalence from more common conditions such as rheumatoid arthritis (0.5–1% prevalence in western populations [1]) and psoriasis (2% prevalence in North America [2]), to much rarer conditions such as Behçet’s disease (estimated 0.005% prevalence in the US [3]). Overall, around 5–7% of the population of western societies has at least one IMID [4], with co-occurrence of multiple IMIDs common [5]. IMIDs are associated with significant, chronic, morbidity affecting quality of life and leading to premature death. As many IMIDs develop later in life, the prevalence is likely to increase as the world population ages.

Despite substantial progress in treatment of IMIDs with newly developed disease-modifying anti-rheumatic drugs and biologics, a substantial proportion of patients fail to respond to treatment or eventually relapse after successful treatment [6]. Consequently, a considerable number of new drugs are in the clinical development pipeline [7] that require demonstration of efficacy and safety. Additionally, with the number of treatments currently available, there is substantial scope for optimising present use through the development of ‘treat-to-target’ approaches [8] and the tailoring of treatment according to patient subgroups [9]. Any such optimised approach also requires demonstration of efficacy and safety, however.

The highest form of evidence is generated by randomised controlled trials (RCTs). For a new drug they provide the most compelling confirmation of benefit over standard therapies. For comparing different treatment optimization strategies, RCTs avoid biases that may occur in an evaluation via a retrospective or prospective observational study. Despite the benefits of RCTs, there are important drawbacks too. RCTs are very expensive to conduct, especially large phase III trials with longer-term follow-up [10]. Accordingly, there has been a strong focus on developing innovative methods for increasing the efficiency of clinical trials. These may have the aim of providing more information from the same number of patients (e.g., by increasing the power to find significant treatment effects), or to reduce the average number of patients recruited to trials without sacrificing power.

In this paper we provide an overview of several innovative methods for increasing the efficiency of clinical trials, framing our discussions within the context of potential benefits to IMID research. We also present a review of recently published IMID trials to investigate how often these approaches have been used in practice.

## Overview of innovative methods for immune-mediated inflammatory disease trials

### Emulating trials from observational data

Given the large costs associated with prospective RCTs, an important question to consider is whether one is needed to answer a research hypothesis. This question has received particular attention in recent years, given the increasing amount of routinely collected data available, from sources such as CALIBER [11]. Furthermore, there are now an array of patient cohorts and registries, with IMID-Bio-UK [12] an example of a UK initiative to bring these together for various IMIDs.

These data sources allow comparisons of different treatment strategies to be conducted through retrospective observational studies. Results from such analyses can be valuable, but are subject to confounding and other flaws such as selection bias and immortal-time bias [13]. This is especially true if inappropriate analyses are applied.

An example, from outside of IMIDs, of where inappropriate analyses gave a misleading answer is presented by Dickerman et al. [14]. The effect of statins on the risk of developing cancer was assessed from retrospective data by comparing individuals who had received multiple years of statin therapy against those who had not. Even after adjustment for potential confounders, this approach was severely biased: a consequence of the fact that individuals who received multiple years of statin therapy could not have done so if they had died from cancer before or during that time. Within IMIDs, a recent paper [15] reviewed retrospective comparative effectiveness evaluations in rheumatoid arthritis; it was found most analyses had some flaws that would potentially lead to biases.

Instead, an approach called emulation of a target trial [16] can address many biases and result in more reliable answers. This involves specifying the ‘target trial’ that one would have liked to have done (i.e., which patient population, intervention, comparator, and outcomes) and analysing the data in a way that emulates this as closely as possible. Each timepoint in the retrospective data is then examined to identify which patients would have been eligible for randomisation in the target trial. The probability that they could have received intervention or comparator is modelled in a way that emulates random assignment from a trial as closely as possible. Dickerman et al. [14] demonstrate how this approach, applied to data from CALLIBER, yields the same conclusions as a large meta-analysis of RCTs for the (lack of) effect of statins on reducing risk of cancer.

With many IMIDs being chronic conditions, RCTs are often used to compare different strategies for employing treatments known to be efficacious. Examples may include testing different ‘treat-to-target’ strategies [8] that may employ more aggressive treatment until a measure of disease activity is below a set threshold. When different strategies are already being employed in practice, and frequent measures of disease activity are recorded in routine data, emulation of target trials may be an efficient approach for evaluating different strategies.

It is important to note, however, that target trial emulation is still subject to bias. This is especially true if the routine dataset does not record sufficient information on potential confounding variables (or if there is a lot of missing data). Consequently, there may still be a need for prospective RCTs of treatment strategies. Nonetheless, target trial emulation could play an important role in prioritising which strategies should be tested and whether an RCT is likely to be successful in finding a significant effect.

### Adaptive trial designs

An adaptive design is one “that offers pre-planned opportunities to use accumulating trial data to modify aspects of an ongoing trial while preserving the validity and integrity of that trial” [17]. Adaptive designs consist of a wide range of approaches that can improve efficiency in trials. Unlike the other innovative methodologies we discuss here, they have been discussed at length in other recent articles. There are both papers that have provided an overview of adaptive designs in general [18] and for specific clinical areas such as rheumatology [19]. We refer the reader to these articles for a comprehension introduction to adaptive designs.

However, we do provide in Table 1 a brief summary of several available types of adaptation and their potential advantages. We also highlight one key factor that influences the added efficiency provided by an adaptive design: the ratio between the recruitment length of the trial and the time taken to observe the primary endpoint [20]. If it takes a long time to observe the primary endpoint, then at an interim analysis there will be a proportion of patients who do not contribute information and who don’t benefit from an adaption. As an example, if the primary outcome takes 1 year to observe and all patients are recruited in 6 months, then by the time the first patient’s one-year outcome has been observed, all patients have been recruited and the adaptive design cannot provide any utility. A more quickly observed ‘intermediate’ outcome can be used to make adaptations, but it must be sufficiently informative for the primary outcome to be useful.

**Table 1:** An overview of various types of adaptive design and their benefits

Adaptive design	Description	Benefits
Group-sequential	Allows a trial to be stopped early for efficacy, futility, or safety, when there is enough evidence to justify doing so.	On average, the sample size that would be required by a trial is reduced; particularly for those with strong treatment effects.
Response adaptive randomisation	Allows the treatment allocation ratio(s) to be altered as the trial progresses.	Allocation can be skewed in favour of the treatment arm that appears to have higher efficacy; meaning more patients are expected to respond in the trial.
Multi-arm multi-stage (MAMS)	Allows multiple treatments to be evaluated in a single trial. Interim analyses allow less promising treatments to be removed from the trial early.	Highly efficient for evaluating multiple treatments at once.
Sample size re-assessment	Allows the sample size to be modified in response to the outcome variation or treatment effect observed in the interim.	The trial is more likely to be powered at the desired level, especially when there is limited data to inform a sample size calculation.
Biomarker adaptive	Allows the trial’s population to be adjusted to avoid enrolling patients who don’t benefit from a treatment; typically this involves incorporating information from, or adapting on, a biomarker.	Patient subgroups who will benefit most from particular treatments can be identified and prioritized.
Platform trial	Allows treatments to be added in to an ongoing trial. Typically involves several treatments being evaluated under an overarching protocol.	Efficient for evaluating multiple treatments as new ones become available over time.

Given the amount of well-developed methodology now available for adaptive trial design, it is this consideration on the choice of primary outcome and its observation time relative to the anticipated recruitment rate, which we believe may principally influence whether an adaptive approach would provide efficiency advantages for a given IMID trial.

### Basket and umbrella trial designs

Because of rapid advancements in biological and genomic understanding during the past few decades, an increasing number of new therapies are being formulated to target specific molecular or immune aberrations. Given that many IMIDs share common mechanisms, these targeted therapies may perform equally well for multiple distinct IMIDs.

**Fig. 1:**
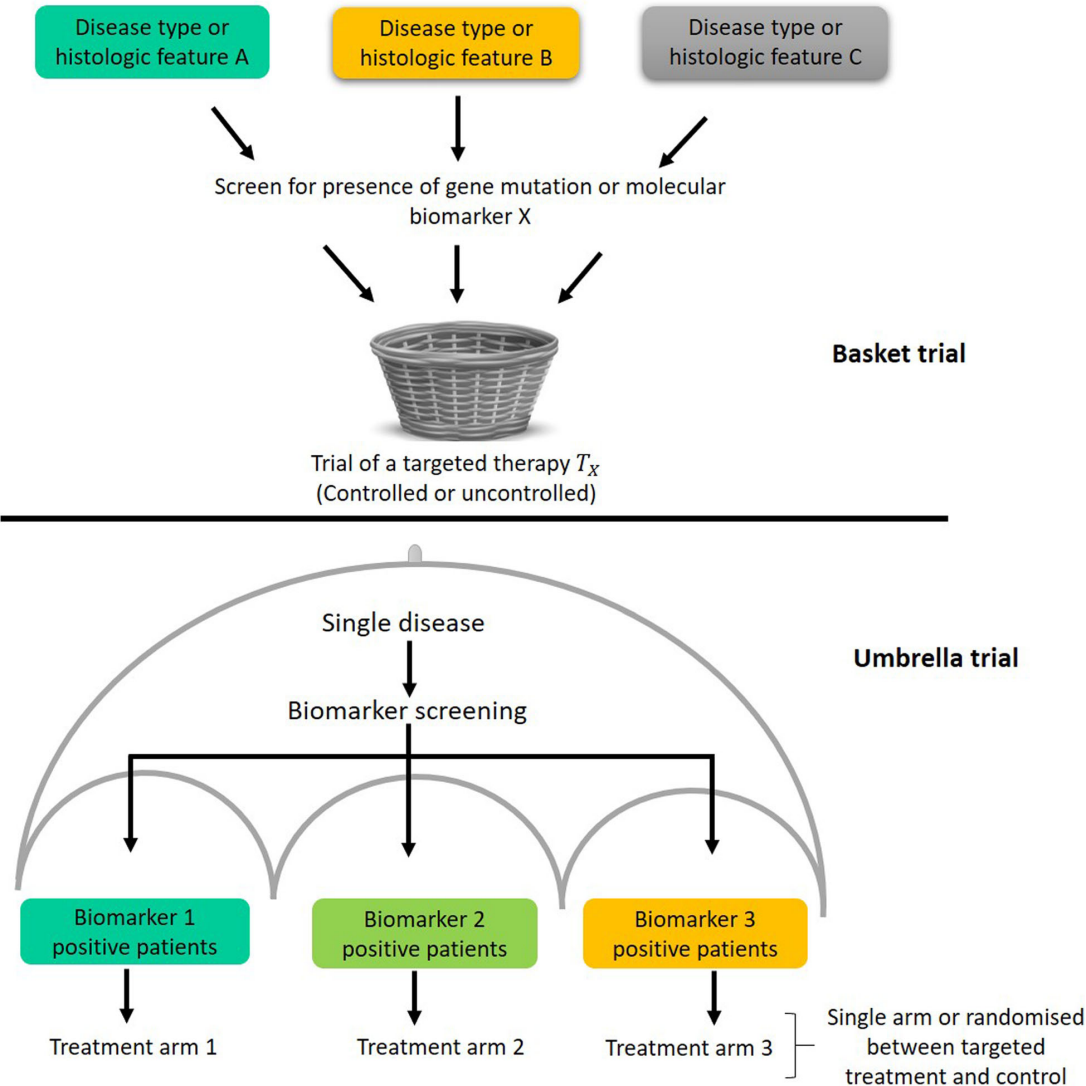
Illustrations of umbrella and basket trial designs, with the sub-studies evaluating the new treatment(s) that are matched by the pre-defined biomarker(s) or genetic mutation(s)

Originating in oncology settings, basket and umbrella trial designs have recently emerged as new types of efficient approaches for testing treatment efficacy in potentially heterogeneous subgroups [21]. These novel designs are administratively efficient as they investigate multiple treatments or diseases, sometimes both, in a single study under an overarching protocol. Figure 1 gives conceptual illustrations of basket and umbrella trial designs with components (sub-studies) defined by biomarkers or genetic mutations, to which the new treatment(s) for evaluation are matched.

While traditional oncology trials focus on a single treatment for a specific cancer histology, basket trials can involve multiple histologies and enrol patients with a common mutation that the new therapy targets. As shown in Fig. 1, an oncology basket trial consists of a number of sub-studies, with each specific to a histology or disease subtype. The prinical aim is to test the treatment efficacy in various sub-studies simultaneously. As examples, Drilon et al. [22] evaluated the efficacy of Larotrectinib, a tropomyosin receptor kinase inhibitor, in diverse TRK fusion positive tumours. Hyman et al. [23] evaluated the BRAF inhibitor vemurafenib, finding significant activity in some tumours (e.g., non-small cell lung carcinoma (NSCLC) and Erdheim-Chester disease), yet inactivity in pancreatic cancer and multiple myeloma.

Efforts have been made to translate the idea of basket designs to disease areas outside of oncology. For example, patients can be stratified to enter a trial with multiple sub-studies by biological characteristics, such as disease stage, number of prior therapies, specific genetic/epigenetic changes, or demographic characteristics [24]. There is also precedent for a basket-type approach having been used in IMID research. Although not officially labelled a basket trial, TRANSREG [25] is a multicentre open-label trial involving 11 IMID patient subgroups evaluating the safety, biological and clinical effects of low-dose interleukin-2. The broad eligibility criteria allow patients with rare IMID diseases to participate in the trial.

Early strategies for analysing basket trials regard the sub-studies in isolation. Although this fully acknowledges the heterogeneity between responses to the same treatment observed in the various patient subgroups, this inevitably leads to low-powered tests due to small sample sizes. Several sophisticated approaches have been developed to enable sharing of information across sub-studies [26–29], among which the proposal by Zheng and Wason [26] can be readily applied to non-oncology basket trials with covariates. With necessary extension or modification, these approaches could lead to the efficient design and analysis of IMID basket trials.

By contrast, umbrella designs, illustrated in Fig. 1, offer the possibility to efficiently test multiple targeted therapies in a single disease population [24]. To date, umbrella designs have only been implemented in oncology [30]: patients of the same tumour type, as screened by an array of biomarkers, receive the treatment specific to their genetic aberration. The ongoing ALCHEMIST trial [31] represents an early example of an umbrella trial. It enrols NSCLC patients and evaluates therapies targeting two types of genetic changes, EGFR mutations and ALK translocations, which are hypothesised as key factors to tumour growth and disease progression.

The increased understanding in pharmacogenomics and pharmacogenetics of IMIDs, especially rheumatoid arthritis [9, 32], makes umbrella designs a suitable approach to answering more treatment-related questions efficiently in a single trial. The identification of specific genes and epigenetic changes involved in the development of rheumatoid arthritis, which may be predictive of the response to treatment, could potentially lead to the initiation of an umbrella trial.

With the multi-biomarker approach of umbrella trials, more patients are likely to meet eligibility criteria for at least one of the biomarker-defined subgroups. This is particularly beneficial compared to an alternative ‘enrichment’ trial that tests one targeted treatment in a subgroup. However, there are unresolved issues in how best to allocate patients who test positive for more than one biomarker, or to no biomarker, in an umbrella trial. Allocating the most suitable treatment to such patients is not straightforward.

Umbrella designs are flexible and can possibly be integrated with various adaptive designs to make them more efficient. Biomarker adaptive randomization could be incorporated to assign patients to the most promising biomarker-linked treatments using accruing trial data (e.g., as in the recent BATTLE trials [33]); a MAMS type approach could be used when a number of treatments are available for evaluation within a cohort; and if promising treatments unavailable at the start of the trial become available, protocol amendments could be made to allow addition of trial arms.

Ultimately, both basket and umbrella designs allow investigators to test more research questions in the same trial. Basket trials help assess whether a new therapy works in distinct patient subgroups (or related diseases) and to what extent [34], while umbrella trials identify whether biomarker-treatment pairs are valid and which one(s) can best improve outcomes.

### Sequential multiple assignment randomised trial (SMART) designs

Therapy of chronic conditions or rapidly fatal diseases often requires several lines of treatment with different drugs or interventions used as the disease progresses. In each line, the treatment may achieve the required clinical objective (e.g., response), or not (e.g., non-response). When treatment fails for a patient at a certain line, it is common medical practice to switch to a different treatment or strategy for the next line. The type or dose of the treatment/intervention may be adjusted repeatedly according to a patient’s ongoing clinical information, including their treatment history and response to previous treatments [35, 36].

**Fig. 2:**
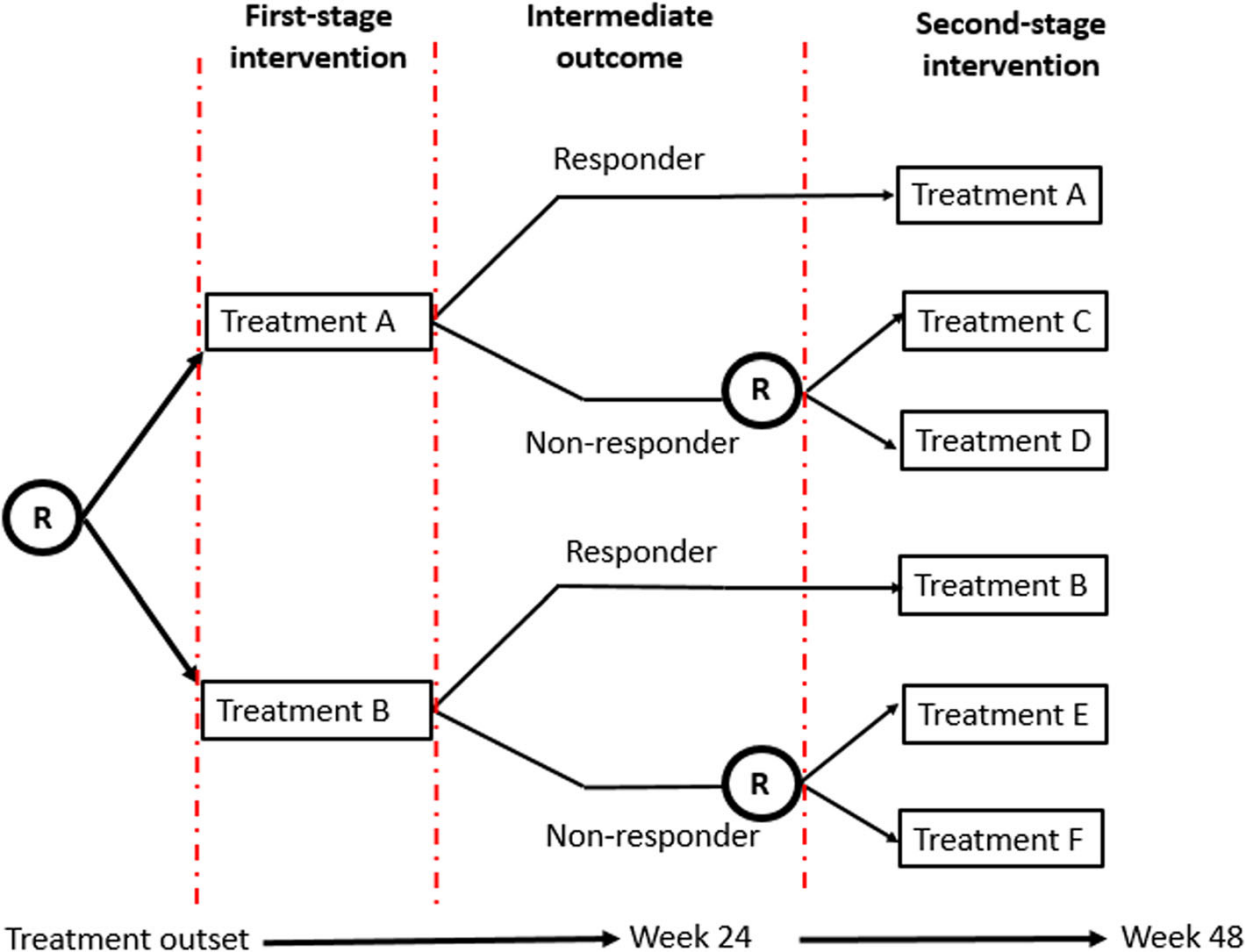
An example SMART design. Only non-responders to the initial treatment are re-randomised in the second stage. R = randomisation

An adaptive intervention is a treatment strategy that personalises treatment through established decision rules that recommend when and how the treatment changes, taking into account the history of previous treatments and response to those treatments [37]. A Sequential Multiple Assignment Randomised Trial (SMART) is a multistage trial design that is used to construct effective dynamic treatment regimens (DTR), also known as adaptive interventions (AIs) or adaptive treatment strategies [38]. Figure 2 depicts an example of a SMART design in which only non-responders to first stage intervention are re-randomised in the second stage. This would provide information to inform an AI that chooses which first-line intervention to use, and how to subsequently treat patients who do not respond to the first-line treatment.

An AI consists of four key elements: critical decision point(s), intervention component(s), tailoring variable(s), and decision rule(s). The first element, a sequence of *critical decision point(s)*, comprises the intervention to begin with, when and how to measure signs of response/nonresponse, how to maintain the success of the initial intervention, and what interventions may be used for non-responders. The second element, the *intervention components,* is a set of intervention/treatment options at each critical decision point. From Fig. 2 we can see that there are two treatments options in the first stage (treatment A and B), and six treatment options in the second stage (two options for responders, and four options for no-responders). The third element is the *tailoring variable(s).* A tailoring variable is an early indicator of the overall outcome (success or failure of the intervention). The response status at week 24 plays the role of the tailoring variable in the example shown in Fig. 2. Lastly, the *decision rules* occurring at each critical decision point link the tailoring variable(s) to the intervention components. Each stage in a SMART corresponds to one of the critical decisions involved in the adaptive intervention. Each participant moves through the multiple stages, and at each stage the participant is randomly (re) assigned to one of several intervention options [35, 39]. Each AI can be summarized in the form (X1;X2:X3) where X1 is the recommended first-stage treatment, X2 the recommended second-stage treatment for responders, and X3 the recommended second-stage treatment for non-responders. There are four different adaptive interventions embedded in the SMART depicted in Figure 2: (A,A,C),(A,A,D),(B,B,E), and (B,B,F).

SMARTs have been used for a wide range of chronic conditions, including some IMIDs. Recent studies that have used them include the CATIE study of treatments for schizophrenia [40], the EXTEND trial of treatments for alcohol dependence [41], and studies of treatments for metastatic renal cell carcinoma [42], depression [43], HIV infection [44, 45], ulcerative colitis [46], autoinflammatory recurrent fever syndromes [47], psoriasis [48–50], and rheumatoid arthritis [51].

An alternative design to a SMART study is the use of “multiple one-stage-at-a-time” randomised trials. This design considers each critical decision point as an independent trial [39]. For instance, from the SMART in Figure 2, there are three different “one-stage-at-a-time” trials. The first trial would correspond to the first stage treatment options. The second trial would study treatment in non-responders to treatment A, and the third trial would study treatment in non-responders to treatment B. One advantage of the SMART design over the “multiple one-stage-at-a-time” is that it uses information from all stages to find the best AI. To do this, it uses Q-Learning; a multistage regression method that can use data from a SMART study to examine whether and how certain variables are suitable to develop an AI or improve an existing one [52, 53].

SMARTs are not without limitation, however. In particular, some issues arise from modelling data from SMARTs when the estimation of the optimal AI is of interest. These include model building, missing data, statistical inference, and choosing an outcome when only non-responders are re-randomised [36]. The fact that the re-randomisation depends on the evolving patient status, along with the sequential design nature of the SMART, bring more complexities to the handling of missing data compared to classical clinical trials. For instance, in a SMART study where only non-responders are re-randomised at the second stage, a patient who is lost to follow-up during the first stage will have missing information on their intermediate response status, second stage treatment, and outcome. It is not possible to know whether the information in the second stage is truly missing or is missing by design since it depends on an unobserved patient response status. Furthermore, the use of flexible regression approaches to avoid complex functions in the Q-learning approach can also make it difficult to acquire interpretable results and valid statistical inference due to potential high variability [36].

SMARTs provide a lot of potential utility to chronic IMIDs, where the most suitable AI is of interest.

### Use of high-dimensional data to stratify patients: adaptive signature trial designs

It is common in clinical trials that only a subgroup of treated patients may benefit from an experimental therapy [54–57]. Identifying these subgroups would allow tailoring of treatment, avoiding costly or toxic treatment of individuals who will not benefit. To identify such subgroups, predictive biomarkers are required. Predictive biomarkers are biomarkers (objective characteristics associated with some aspect of a patient’s function or health), measured at baseline, that are associated with the response to treatment. If a predictive biomarker has been identified, this can be used to predict the likely response to treatment. Some clinical areas, such as oncology, have strong availability of predictive biomarkers. For example, the RAS-mutation identified a subgroup of patients with a significant benefit across all efficacy endpoints associated with treatment for colorectal cancer [58].

However, predictive biomarkers are lacking for most IMIDs, meaning predicting response to treatment is more difficult [59–61]. For example, in rheumatoid arthritis although genetic variants associated with response to methotrexate have been identified [62–65], there is a lack of consensus on the predictive utility of these variants.

In the absence of predictive biomarkers, alternative methods that utilise high-dimensional information could be used. With the rapid development of new next generation sequencing, proteomics, and medical imaging technologies, a large amount of high-dimensional data about patients is starting to be collected in clinical trials. There is the potential for this information to be informative for identifying subgroups of patients who are likely to benefit from a new treatment.

To utilize high-dimensional information in RCTs, a method has been developed known as the adaptive signature design (ASD). The aim of the ASD is to allow a single RCT to both test the overall treatment effect in all patients and to form a predictive biomarker signature that predicts a subgroup of patients who strongly benefit from the treatment. Although the ASD has ‘adaptive’ in its name, it is not actually an adaptive design as it does not change anything about the trial.

**Fig. 3:**
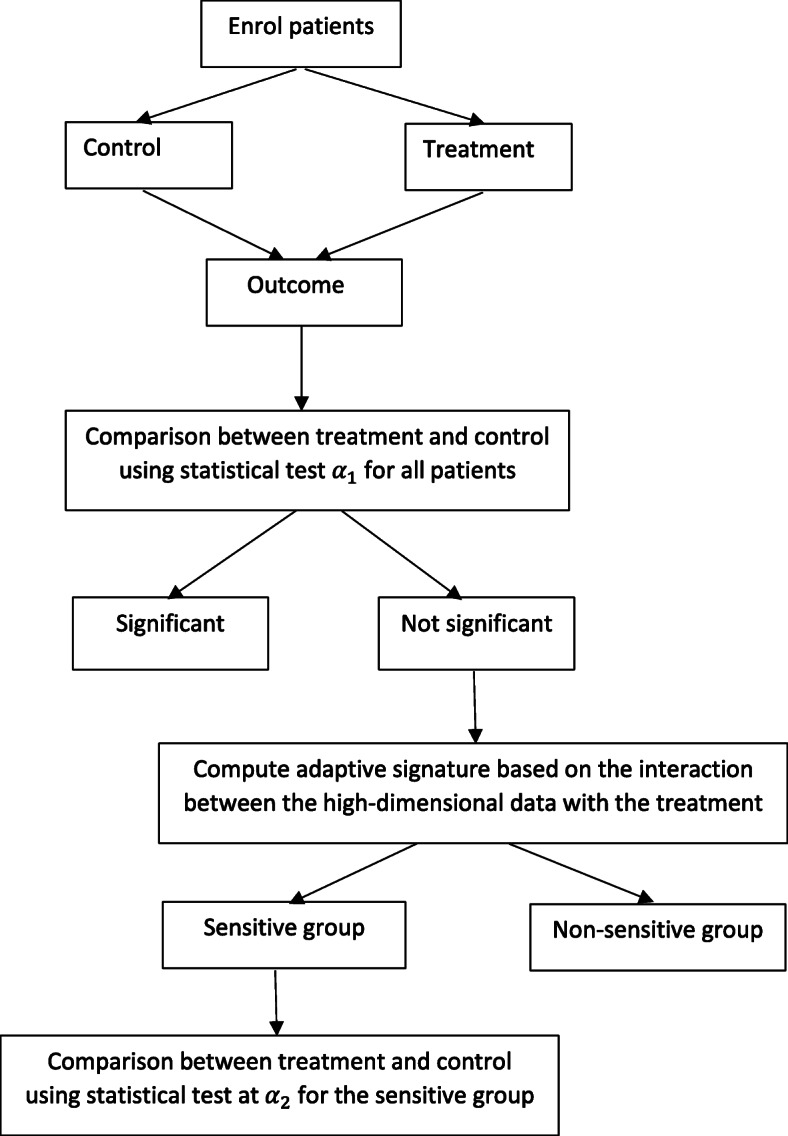
Schematic representation of the adaptive signature design

The original method [66, 67] utilised (high-dimensional) gene expression data in an oncology setting, but it can be used in any case where heterogeneity in the treatment effect is expected and there is high-dimensional information available. Which of the high-dimensional data should be included in the signature is determined by imposing a threshold on the significance level, odds ratios, and number of biomarkers. Further papers have proposed modifications of the original ASD [68–70] to provide improved performance (in terms of correctly identifying a subgroup who benefit from treatment). In these methods, the high-dimensional data is used to form a signature that is computed based on the interaction between these data with the treatment. The adaptive signature is represented by a single score for each patient. The scores can then be utilised to divide the patients into subgroups using a variety of clustering techniques, or as covariates in the tests of association with the outcome. The test for the overall comparison between the arms can be performed by testing for the difference between the arms in the trial population (at the significance level α_1_) and testing for the difference between the arms in the subgroup (at significance level α_2_). The overall significance level of the trial is then controlled at the α = α_1_ + α_2_ level (Fig. 3).

In conclusion, ASDs are a novel methodology that can develop and validate predictive signatures in a single trial. They have the potential to increase the efficiency of clinical trials by finding the group of patients benefiting from particular treatments. However, when the clinical benefit for a subgroup is minimal, a large sample size might be required to detect it with sufficient power. Additionally, the performance of the designs deteriorates if there are many covariates that are not associated with patient benefit. To address this issue, an additional pre-filtering of the covariates might be required. This family of designs may also benefit from exploring different methods of interaction of treatment with high dimensional covariates [71, 72], and from considering multiple trial endpoints [73]. These considerations notwithstanding, ASDs offer a potential route to identifying patient subgroups that will benefit from treatment in IMIDs for which predictive biomarkers are currently lacking.

### Composite responder endpoints and augmented analysis methods

Clinical trials specify primary and secondary outcomes that measure how patients respond to a treatment or intervention. The primary outcome should be chosen as a measurement that will be more favourable if the treatment being tested is efficacious or effective. As many IMIDs have complex manifestations and multiple symptoms, it can be difficult to specify a single measurement as being the most important. For this reason, it is common that primary outcomes in IMID trials combine multiple relevant measurements into a single composite outcome. A specific type of composite endpoint is a responder endpoint, which divides patients into responders and non-responders based on different measurements, or components. Some of these components can be binary and others may be whether continuous measurements are above a threshold.

The standard method of analysis for composite responder endpoints is to treat them as binary variables (responder or non-responder). The analysis then estimates the proportion of patients who are responders and whether there is a significant difference between arms: this is done with a suitable binary method such as Fisher’s exact test or logistic regression, amongst many others.

Responder endpoints have the appealing property of summarising very complex information into an easy-to-interpret single quantity. This is also a limitation when applying analysis methods that treat the outcome as binary: much information is discarded, especially from continuous components when dichotomising (see, e.g. [74, 75]) which can lead to a reduction in power [76].

Assuming that the responder endpoint is clinically relevant, there are alternative ways of estimating the proportion of patients who are responders. For endpoints that define response based on a single continuous component, methods were proposed in the 1990s to more precisely estimate the proportion of responders [77, 78]. For composite responder endpoints that are a mixture of continuous and binary components, the augmented binary method has been proposed to provide higher efficiency. This was originally proposed for response criteria endpoints used in phase II oncology trials [79] but has since been extended to endpoints used in IMIDs such as rheumatoid arthritis [80] and systemic lupus erythematosus (SLE) [81]. The method has also been extended to endpoints that are formed from the time until a composite event occurs [82] (e.g., time until relapse, where relapse involves a continuous biomarker being above a certain level), although further work in this area is needed.

The augmented binary method requires no additional data to be collected; it simply fits a more complex statistical model to the data collected on the different components and uses this model to estimate the difference between arms in the proportion of responders (together with a confidence interval and *p*-value). It has been shown in various papers [80, 81, 83, 84] to provide large gains in efficiency, equivalent to applying the traditional binary analysis with a sample size of 30% or more higher. The extent of the increase of efficiency depends on to what extent the continuous component(s) distinguish between responders and non-responders [85].

A previous review [86] found that several IMID conditions used composite responder outcomes. We show some examples of these in Table 2.

**Table 2:** Examples of composite responder endpoints used in IMID trials

IMID	Endpoint	Definition
Ankylosing spondylitis	ASAS20 response	• 20% improvement and ≥ 10 units of change (on a 0–100 scale) in each of 3 domains • No worsening of a similar amount in the fourth domain • (Components are physical function, pain, inflammation and patient’s global assessment)
Crohn’s disease	Clinical remission	• Crohn’s Disease Activity Index below a threshold (e.g., 150) • No use of steroids or rescue treatment
Idiopathic arthritis-associated uveitis	Best corrected visual acuity above threshold and no light perception	• Best-corrected visual acuity, thresholds ≤20/50, ≤20/200 • No light perception •Contribution of amblyopia, yes/no
Juvenile arthritis	Response	Improvement by 30% in at least 3 of: • MD global assessment • parent or patient global assessment • functional ability • number of joints with active arthritis • number of joints with limited range of motion • Erthrocyte Sedimentation Rate
Juvenile dermatomyositis	Responder index	• ≥4 point reduction from baseline in safety of estrogen in lupus national assessment (SELENA) systemic lupus erythematosus disease activity index (SLEDAI) score • No worsening (increase of < 0.30 points from baseline) in physician’s global assessment (PGA) • No new British Isles Lupus Assessment Group of SLE clinics (BILAG) A organ domain score or 2 new BILAG B organ domain scores compared with baseline
Nonalcoholic steatohepatitis	Resolution of steatohepatitis without fibrosis	• Improvement in NAS of two points • No worsening of fibrosis
Sjogren’s syndrome	Response	• > 30% reduction in analog scales evaluating dryness, pain and fatigue

## Current use of innovative methods in immune-mediated inflammatory disease trials

### Review methods

To investigate the frequency with which innovative methods have been used in IMID trials in recent years, we searched PubMed on June 182,020. We restricted our evaluation to clinical trial publications that have appeared since 2018 in any of five high impact factor journals relevant to IMIDs (*New Engl J Med*, *Lancet*, *Ann Rheum Dis*, *Arthritis Rheumatol*, *J Am Acad Dermatol*). To provide a comprehensive evaluation, we included articles containing any of 51 IMID disease terms. See the Supplementary Materials for the search term. This search returned 160 articles for review.

Each article was reviewed by JMSW to establish whether it met the inclusion criteria: that the article was a primary report of the results of a clinical trial conducted to evaluate the efficacy of one or more treatments for one or more IMIDs. Retrospective trial analyses were thus excluded, as our focus was on how innovative methods have been used in practice in the design and analysis of IMID trials. For each article deemed eligible for inclusion, data was extracted by JMSW for 21 questions relating to the trial’s design and analysis, and in particular the use of innovative methods (see Supplementary Table 1). Owing to the objective nature of the extraction questions, high reproducibility on evaluation of inclusion and subsequent data extraction was anticipated. Nonetheless, ten articles were randomly chosen for duplicate review by MJG. The authors agreed on inclusion for all ten articles. Agreement on extracted data was 95%. See the Supplementary Materials for further details.

### Findings

Ninety-seven articles were deemed to be eligible for inclusion. A summary of the extracted data for these 97 articles is given in Table 3.

**Table 3:** Summary of extracted data for the 97 included articles. The denominator for computing percentages (given to 1 decimal place) is 97 unless stated otherwise

Question	n (%)
What immune-mediated inflammatory disease(s) was the trial conducted in?^a^	
Rheumatoid arthritis	30 (30.9)
Systemic lupus erythematosus	10 (10.3)
Psoriasis	9 (9.3)
Psoriatic arthritis	9 (9.3)
Juvenile idiopathic arthritis	4 (4.1)
Multiple sclerosis	4 (4.1)
Sjögren’s syndrome	3 (3.1)
Systemic sclerosis	3 (3.1)
Other (see Supplementary Materials)	25 (25.8)
How many treatment arms were in the trial?	
1	5 (5.2)
2	63 (64.9)
3	13 (13.4)
4	11 (11.3)
5	2 (2.1)
6	3 (3.1)
What was the total planned sample size according to the sample size calculation?	Median: 214 IQR: [113, 400] Range: [10, 5400]
How was the trial funded?	
Industry	73 (75.3)
Academic	16 (16.5)
Mixed	4 (4.1)
Not reported	3 (3.1)
Charity	1 (1.0)
Was any innovative design used?^b^	
Yes	19 (19.6)
Group-sequential design/futility interim analysis	7 (7.2)
Sequential multiple assignment randomised trial design	6 (6.2)
Bayesian methods used	4 (4.1)
Sample size re-estimation	2 (2.1)
Basket trial design	1 (1.0)
Use of an innovative design by trial funding	
Industry	16/73 (21.9)
Other	3/24 (12.5)
Did the trial design report the involvement of any evidence from routinely collected data, cohorts, or biobanks?	
Yes	2 (2.1)
What was the length of patient recruitment (in weeks)?^c^	Median: 96 IQR: [55, 120] Range: [16, 296]
What was the primary endpoint timepoint (in weeks)?^d^	Median: 24 IQR: [12, 48] Range: [4, 240]
Did the exclusion criteria explicitly include the presence of another autoimmune disease?^e^	
Yes	58 (59.8)
Were any endpoints based on dichotomizing continuous information?	
Primary	66 (68.0)
Secondary	81 (83.5)
How were dichotomized responder endpoints analysed?^f^	
Cochrane-Mantel-Haenszel test	27 (27.8)
Logistic regression	21 (21.6)
Chi-square test	9 (9.3)
Cox model	9 (9.3)
Fisher’s exact test	9 (9.3)
Log rank	5 (5.2)
Other	16 (16.5)
Any high-dimensional data collected at baseline (gene expression, GWAS, synovial biopsies etc.)?^g^	
Yes	8 (8.2)

^a^A small number of articles included patients with more than one IMID in their trial, though none for the diseases named here

^b^One article used a sequential multiple assignment randomised trial design with an interim futility assessment

^c^One article did not report the recruitment period. To translate recruitment periods given inmonths to weeks, 4 weeks was taken to be equivalent to 1 month

^d^For two, one, and one article respectively the median, mean, and maximum follow-up times are used. To translate recruitment periods given in days and months to weeks, 30 days and 1 month were taken to be equivalent to 4 weeks

^e^One article reported “any serious illness” as an exclusion criteria and is considered as ‘No’ for the extraction

^f^Articles may have utilised more than one method

^g^Three articles that collected MRI imaging data, for which it was unclear as to whether this was high-dimensional, are listed as ‘No’ for the extraction

While more than 20 distinct conditions were evaluated in the eligible trials, the plurality (31%) found were in rheumatoid arthritis. Notable numbers were also found in psoriatic arthritis, psoriasis, and SLE. The majority of trials (75%) were funded and sponsored by industry.

Most (65%) eligible trials had two arms. Some rarer conditions used single arm trials with no prospective control arm. In other cases, more than two arms were included: in most instances this was for industry-funded trials of a new drug, with different doses or regimens included as distinct arms. We did not identify any MAMS trials.

There was some reported use of innovative approaches (19.6%). These consisted predominantly of group-sequential designs (or a futility analysis), sample-size re-assessment, and re-randomising some participants as in a SMART design. For re-randomisations, we did not find any examples where an analysis was performed to determine the best AI. The median recruitment length was 96 weeks and primary endpoint length was 24 weeks. This indicates that for a majority of trials the ratio of endpoint length to recruitment length would be sufficiently low for an adaptive design to provide efficiency [20].

In a majority of trials (60%), patients with other autoimmune diseases were not eligible for the trial. In other cases, this was not an explicit exclusion criteria but it is likely that such patients would be indirectly excluded through criteria such as being naïve to therapies that are commonly used for other IMIDs.

We found very few examples where collection of high-dimensional data was reported (8.2%). In the eight trials that did report this, the most common approach was to analyse each variable separately. Reported use of routinely collected data in the design of the trial was also low.

The use of responder endpoints (involving dichotomization of continuous measurements) was very high. The majority of trials (68%) had a primary endpoint that was defined in this way; an even higher proportion (84%) had a responder endpoint as a secondary outcome. These endpoints were routinely analyses using standard methods, such as a Cochrane-Mantel-Haenszel or Fisher’s exact test.

### Use of innovative methods in currently ongoing trials

There is often a long lead time between designing a trial and it being reported. We therefore also conducted a scoping review of use of innovative designs in trials that are currently underway. We searched clinicaltrials.gov on 2 February 2021 for studies that were ‘not yet recruiting’, ‘recruiting’, ‘enrolling by invitation’, or ‘active, not recruiting’ that contained any of 51 IMID disease terms and any of 39 terms related to innovative design. A link to conduct this search is given in the Supplementary Materials. It returned 49 studies that were then reviewed by MJG to evaluate evidence of innovative design use.

There were some examples of innovative designs being used. This included multiple group-sequential and seamless phase II/III trials. We also found trials using a Bayesian basket design (NCT04498962), MAMS design (NCT03092674, NCT03805789) and several uses of adaptive randomization (NCT04596293, NCT02269280, NCT02593123). With limited details provided in trial registrations compared to trial publications, it was not possible to extract detailed information and we may well have missed use of innovative approaches.

## Discussion

In this paper we have provided an overview of innovative methods that could provide utility to IMID trials. These methods and their advantages are summarized in Table 4. We have also shown that few recently reported trials are utilizing innovative approaches through a literature review.

**Table 4:** Summary of innovative design and analysis approaches briefed in this paper

Innovative method	Summary
Adaptive designs	• Offer opportunity to make changes to the design of an ongoing trial as patient outcome data is accrued. • Can improve efficiency of trial (more power for same sample size, or reduced sample size for same power), make trial more robust to design assumptions, and/or improve patient benefit provided by trial. • Benefit relies on the primary endpoint (or informative intermediate endpoint) being observed relatively quickly compared to recruitment.
Adaptive signature design	• Uses high-dimensional data to form a ‘sensitive’ subgroup of patients who experience higher benefit from an intervention in comparison to the overall population. • Allows forming, and confirmatory testing, of a predictive signature in the same trial. • May be difficult to interpret the resulting signature.
Augmented analysis of composite responder outcomes	• Efficiently analyse responder endpoints, which classify patients as responders or non-responders on the basis of a combination of binary and continuous measurements. • Can substantially improve the power of trials using responder endpoints whilst maintaining the clinically relevant outcome. • More complex analysis that makes extra assumptions compared to the traditional analysis approach.
Basket and umbrella designs	• Use an overarching protocol to test interventions in related disease conditions or patient subgroups, simultaneously. • Allow operational and statistical efficiencies; with the latter realised by using advanced statistical approaches that can e.g., share information between the different arms of the trial. • Generally requires assuming the same endpoint and control group, despite various sub-studies, in the trial.
Emulation of trials	• A method for using large retrospective datasets to predict what it would have been if yielded by a randomised controlled trial. • Exploits the value of data that is already collected. • Analysis makes strong assumptions and can only compare interventions in current use.
Sequential Multi Assignment Randomised Trials (SMART)	• Allow multiple randomisations of patients at different stages of the study. • Allow separate research questions to be answered and for the optimal ‘adaptive intervention’ to be found. • For a specific AI, they allow to improve individual outcomes by further tailoring treatment by baseline or time-varying characteristics.

Although 19.6% of included trials used some approach that we classified as innovative, most of these were relatively straightforward approaches, such as a futility analysis or having a second randomization of non-responding patients (without applying techniques for analysing SMARTs.) Assessment of current IMID trials listed on clinicaltrials.gov indicates that use of innovative approaches may still be infrequent. There is a high potential for more advanced innovative approaches to be used in future IMID trials, but this requires improved awareness, education, and software.

One notable finding was that it was very common, amongst multiple distinct IMIDs, for trial endpoints to be responder endpoints. Over two-thirds of trials had such an endpoint as the primary, and almost 90% had a secondary endpoint. In every case the endpoint was analysed as if it were binary. As we have described, there are much more efficient analysis methods available and it is important for them to be made available for use in practice. Some freely-available software is currently available [87] but there is the need for more generic software and methods that can be used across all such endpoints used in IMID trials.

Presently, it appears that collection of high dimensional information and use of routinely collected data is rare in IMID trials. A limitation of our review is that we may have missed use of this from just examining primary reports of RCTs. For example it may be common for high-dimensional information to be collected but reported in secondary analysis papers. In addition it may not be felt a worthwhile use of space in a primary report of an RCT to discuss how routinely collected data was used to inform the trial design.

The majority of trials were sponsored and funded by industry. Although there were uses of innovative approaches in industry sponsored trials, use of more advanced methods that we have discussed in this paper could be hampered by regulatory issues (either actual or perceived). For use of some more advanced designs and analysis approaches in confirmatory trial settings, it will be important to ensure they are supported by regulators.

A final important consideration for the potential applicability of the discussed innovative methods is disease prevalence. Some methods we have discussed are particularly relevant in rare disease settings: 1) As composite endpoints are recommended for rare diseases, the augmented analysis methods are more applicable [88]; 2) Basket trials potentially allow borrowing of information, and may thus improve analysis of related rare IMIDs (or for a rare IMID to be tested in conjunction with a common IMID); 3) adaptive designs may be more relevant in rare diseases due to the need to improve efficiency [89] and can be used in single-arm trials, such as the Simon two-stage design [90] that is widely used in phase II cancer trials [91]. Other approaches may not be so applicable in rare settings due to the need for high sample sizes.

In conclusion, IMID trials could substantially benefit from use of more innovative approaches that we have reviewed in this paper. Further research, better software, and more dissemination is needed to ensure all IMID trials, that could benefit, do so.

## Supplementary Information

**Additional file 1.**

## Data Availability

All data generated or analysed during this study are included in this published article [and its supplementary information files].

## Abbreviations

AI: Adaptive intervention
ASD: Adaptive signature design
DTR: Dynamic treatment regime
IMID: Immune-mediated inflammatory disease
MAMS: Multi-arm multi-stage
NSCLC: Non-small cell lung carcinoma
RCT: Randomised controlled trial
SLE: Systemic lupus erythematosus
SMART: Sequential multiple assignment randomised trial
